# Cross‐cohort epigenome‐wide association study in Alzheimer's disease risk and related traits

**DOI:** 10.1002/alz.093877

**Published:** 2025-01-09

**Authors:** Lidija Milicic, Michael Vacher, Tenielle Porter, Vincent Dore, Pierrick Bourgeat, James D. Doecke, Rosita Shishegar, Paul Maruff, Colin L. Masters, Christopher C. Rowe, Victor L Villemagne, Simon M. Laws

**Affiliations:** ^1^ Edith Cowan University, Perth, Western Australia Australia; ^2^ Edith Cowan University, Joondalup Australia; ^3^ Curtin Medical School, Curtin University, Bentley, Western Australia Australia; ^4^ The Australian e‐Health Research Centre, Commonwealth Scientific and Industrial Research Organisation, Melbourne, VIC Australia; ^5^ The Australian e‐Health Research Centre, Commonwealth Scientific and Industrial Research Organisation, Brisbane, QLD Australia; ^6^ Centre for Precision Health, Perth, Western Australia Australia; ^7^ The Australian e‐Health Research Centre, CSIRO, Parkville, VIC Australia; ^8^ Florey Institute of Neuroscience and Mental Health, Parkville, VIC Australia; ^9^ The Florey Institute of Neuroscience and Mental Health, University of Melbourne, Melbourne, VIC Australia; ^10^ Department of Nuclear Medicine and Centre for PET, Austin Health, Heidelberg, Vic, 3084, Australia, Heidelberg, VIC Australia; ^11^ University of Pittsburgh, Pittsburgh, PA USA; ^12^ Centre for Precision Health, Edith Cowan University, Joondalup, Western Australia Australia

## Abstract

**Background:**

Epigenome‐wide association studies (EWAS) have identified multiple loci that are differentially methylated in Alzheimer’s disease (AD). However, for complex diseases such as AD, single methylation sites associated with disease and disease‐related traits have relatively low effect sizes. At the genetic level, measures of cumulative genetic risk, such as polygenetic risk scores, have yielded success in risk prediction as well as in association and interaction studies. Such approaches have the potential to be directly transferred to DNA methylation data.

**Method:**

This study utilised data from two well‐characterised longitudinal cohort studies, the Australian Imaging, Biomarker and Lifestyle (AIBL) study (n=707) and the Alzheimer’s Disease Neuroimaging Initiative (ADNI; n=620) and aimed to investigate the association between peripheral blood DNA methylation, at CpG sites across the epigenome, and eight AD‐related phenotypes: AD risk, MRI‐derived brain volumetric measures (hippocampal, ventricle, grey and white matter volumes), PET derived brain Aß‐amyloid burden (as a continuous and binary (high/low) variable) and cognition (Pre‐Alzheimer’s Cognitive Composite (PACC)). Each trait‐specific EWAS was first undertaken in the AIBL cohort, and eight trait‐specific methylation risk scores (MRS) were then developed using summary statistics. An additional MRS was also developed using an elastic net machine learning method. MRSs were then validated within and across traits in the ADNI cohort.

**Result:**

Nominally significant associations were observed between CpG sites within several of the EWAS that were performed; however, no individual CpG sites remained significant after FDR correction. Several MRSs were associated with AD risk after correction for the false discovery rate.

**Conclusion:**

While this study adds to the growing literature supporting the role of MRSs in AD‐related traits, it suggests that there are limited associations of individual epigenome‐wide CpG sites with cognitive and neuroimaging traits using the methods undertaken in this study. Future studies with larger cohort sizes and phenotypically rich datasets will help to confirm these findings and potentially elucidate further associations.

